# Atorvastatin but Not Pravastatin Impairs Mitochondrial Function in Human Pancreatic Islets and Rat β-Cells. Direct Effect of Oxidative Stress

**DOI:** 10.1038/s41598-017-11070-x

**Published:** 2017-09-19

**Authors:** Francesca Urbano, Marco Bugliani, Agnese Filippello, Alessandra Scamporrino, Stefania Di Mauro, Antonino Di Pino, Roberto Scicali, Davide Noto, Agata Maria Rabuazzo, Maurizio Averna, Piero Marchetti, Francesco Purrello, Salvatore Piro

**Affiliations:** 1 Department of Clinical and Experimental Medicine, Garibaldi Hospital, University of Catania, Catania, Italy; 2 Department of Clinical and Experimental Medicine, Islet Cell Laboratory, University of Pisa, Pisa, Italy; 3 Department of Biomedicine, Internal Medicine and Medical Specialties (DIBIMIS), University of Palermo, Palermo, Italy

**Keywords:** Mechanisms of disease, Cardiology, Type 2 diabetes

## Abstract

Statins are a class of drugs widely prescribed as frontline therapy for lowering plasma LDL-cholesterol in cardiovascular risk prevention. Several clinical reports have recently suggested an increased risk of type 2 diabetes associated with chronic use of these drugs. The pathophysiology of this effect remains to be fully elucidated but impaired β-cell function constitutes a potential mechanism. The aim of this study was to explore the effect of a chronic treatment with lipophilic and hydrophilic statins on β-cell function, using human pancreatic islets and rat insulin-secreting INS-1 cells; we particularly focused on the role of mitochondria and oxidative stress. The present study demonstrates, for the first time, that atorvastatin (lipophilic) but not pravastatin (hydrophilic) affected insulin release and mitochondrial metabolism due to the suppression of antioxidant defense system and induction of ROS production in pancreatic β-cell models. Mevalonate addition and treatment with a specific antioxidant (N-AcetylCysteine) effectively reversed the observed defects. These data demonstrate that mitochondrial oxidative stress is a key element in the pathogenesis of statin-related diabetes and may have clinical relevance to design strategies for prevention or reduction of statin induced β-cell dysfunction and diabetes in patients treated with lipophilic statins.

## Introduction

Statins are specific, potent and competitive inhibitors of 3-hydroxy-3-methyl-glutaryl-CoA reductase (HMG-CoA reductase), a rate-limiting microsomal enzyme in the biosynthesis of cholesterol through the mevalonate pathway.

The resulting reduction in hepatic levels of cholesterol initiates a series of coordinated reactions in cholesterol homeostasis including the up-regulation of the low density lipoprotein receptor (LDL-R) which in turn leads to enhanced clearance of LDL-particles from the blood^1, 2^. In addition to the lipid lowering effect, pleiotropic properties of this class of drugs have been identified, such as improvements of endothelial function, stabilization of atherosclerotic plaques, and anti-inflammatory actions^3^.

In the last few years, a growing body of evidence has highlighted a 10–12% increase in new-onset diabetes mellitus (NODM) among patients on statin therapy^4–6^. This issue first came to light in the JUPITER trial (Justification for the Use of Statins in Primary Prevention: An Intervention Trial Evaluating rosuvastatin)^7, 8^; consequently, in March 2012, the US Food and Drug Administration (FDA) decided there was sufficient evidence to support the addition of a warning label about diabetes risk on statin packaging^9^.

The diabetogenic effect of statins seems to be directly related to the dose of statins, to the degree of attained LDL cholesterol lowering^10^ and to the hydrophilic or lipophilic nature of statins which make them more or less hepato-selective, respectively. Indeed, a high hepato-selectivity translates into minimal interference with cholesterol metabolism in tissues other than the liver and consequently in a lesser diabetogenicity^11–13^.

The benefits of statin therapy in reducing cardiovascular (CV) events far outweigh the diabetes hazard^8, 14, 15^; nevertheless, it is important to deeply understand the molecular mechanisms through which these compounds affect glucose homeostasis.

These mechanisms could potentially involve an increased insulin resistance, a decreased β-cell function or a combination of these two processes^16^.

Although statins have proved to be generally well tolerated with a low prevalence of side effects, as the prescription rates have increased, more adverse effects have been identified, with the most common being myopathy^17, 18^. Several studies have shown that mitochondrial impairments could be largely implicated in the onset of this side effect^19–21^, furthermore, recent investigations in skeletal muscle of humans and rats, have demonstrated that increased ROS (reactive oxygen species) production was responsible for the mitochondrial dysfunction observed during statin treatment^22, 23^.

In this study we focused our attention on statin-related pancreatic β-cell impairments and investigated the effect of a chronic (24–48 h) treatment with atorvastatin (lipophilic) or pravastatin (hydrophilic) at different concentrations on insulin secretion and β-cell function using human pancreatic islets and *in vitro* cultured pancreatic β-cells. We specifically focused on these statins since the literature indicates atorvastatin and pravastatin respectively the more and the less diabetogenic statin^6, 24–27^, and also in order to address whether lipophilic (atorvastatin) and hydrophilic (pravastatin) statins exert similar effects. Additionally, because the mitochondrion plays a key role in glucose-induced insulin release and since in the same way as skeletal muscle cells, even pancreatic β-cells are at high risk of oxidative damage, due to the weakness of ROS-scavengers, we investigated mitochondrial function and ROS production in models of pancreatic β-cells chronically treated with statins.

Since the inhibition of the HMG-CoA conversion to mevalonate suppressed not only the synthesis of cholesterol, but also of other intermediates, such as Coenzyme Q10 (CoQ10), a major radical-scavenging antioxidant^19^, we also investigated CoQ10 modulation and mevalonate co-treatment effect in our system.

Finally, to definitely clarify the role of oxidative stress in our model, we tested the effect of a co-treatment with N-AcetylCysteine, (NAC) a well-known radical scavenger.

## Results

### Atorvastatin but not pravastatin affected both basal and glucose-induced insulin secretion in human pancreatic islets and in INS-1 cells

**Figure 1:**
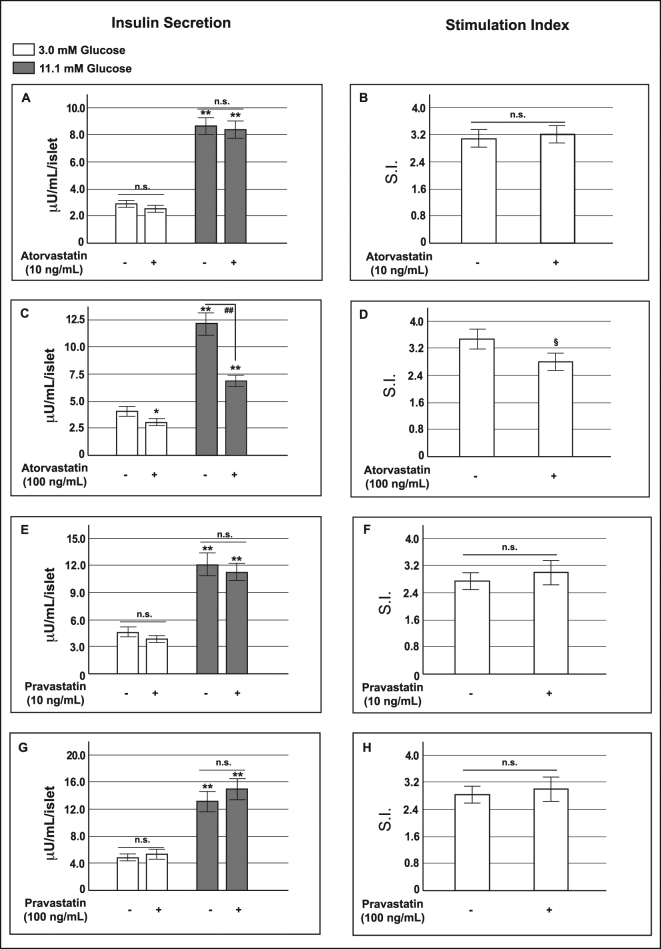
Effect of atorvastatin and pravastatin on glucose-induced insulin release in human pancreatic islets. Absolute glucose-induced insulin secretion (expressed as µU/mL/islet) and relative stimulation index (S.I.) in control human pancreatic islets and in islets pre-exposed for 48 h to atorvastatin 10 ng/mL (Panels A and B) or 100 ng/mL (Panels C and D) and pravastatin 10 ng/mL (Panels E and F) or 100 ng/mL (Panels G and H). *P < 0.05, **P < 0.01 vs. control at 3.0 mM glucose; ^##^P < 0.01 vs. control at 11.1 mM glucose; ^§^P < 0.05 vs. S.I. in control islets; n.s. not significant (1-way ANOVA followed by Bonferroni test, n = 9).

To study the effects of statin treatment on insulin release, we firstly investigated acute glucose-stimulated insulin secretion in human pancreatic islets that had been chronically pre-exposed for 48 h to atorvastatin or pravastatin (10 or 100 ng/mL) (Fig. 1). We used nine different islet preparations, obtained by collagenase digestion and density gradient purification from the pancreas of multiorgan donors (Supplementary Table 1).

Insulin secretion was expressed as absolute value (µU/mL/islet) and as stimulation index (S.I.), i.e. the ratio of stimulated over basal insulin secretion.

As shown in Panel A of Fig. 1, in islets pre-exposed to atorvastatin 10 ng/mL for 48 h, both basal (LG = 3.0 mM) and glucose-stimulated (HG = 11.1 mM) insulin secretion were slightly, but not significantly, decreased with respect to islets exposed to the relative vehicle (corresponding to 10^−6^% DMSO). On the contrary, exposure to the higher dose of atorvastatin (100 ng/mL) significantly reduced the insulin release in response to either low (3.0 ± 0.3 µU/mL/islet; p < 0.05) and high glucose (7.3 ± 0.6 µU/mL/islet; p < 0.01), compared to the relative vehicles (corresponding to 10^−5^% DMSO)(4.3 ± 0.6 µU/mL/islet and 12.2 ± 1.5 µU/mL/islet, at low and high glucose respectively) (Fig. 1, Panel C). As a consequence, the insulin stimulation index (ISI) decreased from 3.4 ± 0.4 in the vehicle-treated islets to 2.8 ± 0.3 in the islets exposed to atorvastatin 100 ng/mL (p < 0.05) (Fig. 1, Panel D).

In contrast, in pancreatic islets that had been pre-exposed to pravastatin both basal and glucose-induced insulin secretion were unaffected for all of the tested dose-time combinations (Fig. 1, Panels E–H).

**Figure 2:**
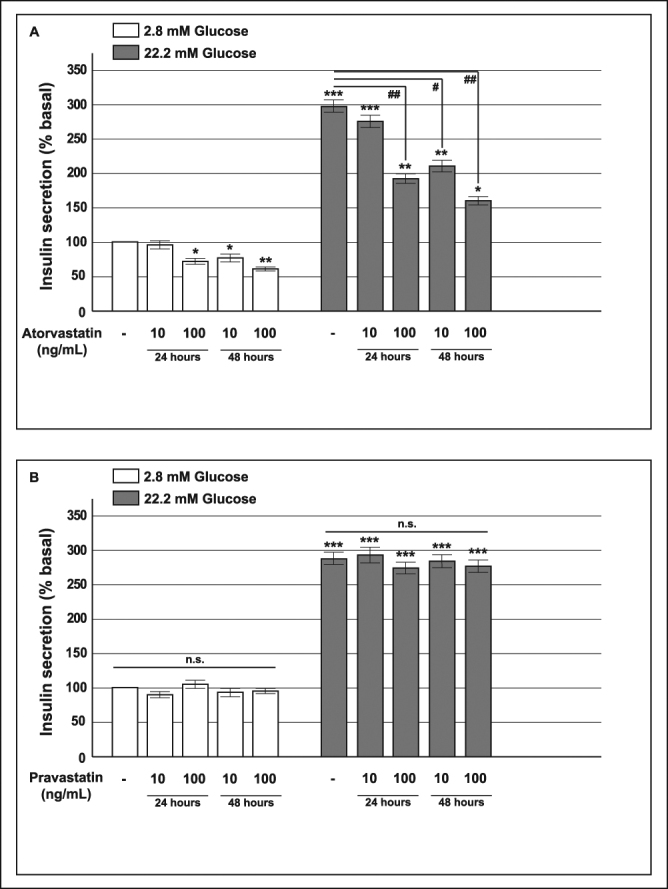
Effect of atorvastatin and pravastatin on glucose-induced insulin release in INS-1 cells. Panel A: acute glucose-induced insulin secretion in control cells and in cells pre-exposed to 10 or 100 ng/mL of atorvastatin for 24 or 48 h (baseline secretory rate at 2.8 mM glucose: 32.3 ± 3.5 ng/mg of protein in 1 h); Panel B: acute insulin secretion in INS-1 cells pre-exposed to 10 or 100 ng/mL of pravastatin for 24 or 48 h (baseline secretory rate at 2.8 mM glucose: 37.1 ± 4.8 ng/mg of protein in 1 h). *P < 0.05, **P < 0.01, ***P < 0.001 vs. control at 2.8 mM glucose; ^#^P < 0.05, ^##^P < 0.01 vs. control at 22.2 mM glucose; n.s. not significant (1-way ANOVA followed by Bonferroni test, n = 4).

To further investigate the effect of statins on insulin release and beta cell function and to ascertain whether the observed effects are direct or dependent upon other islet cell types, we switched to a model that, unlike intact islets, contains only beta cells, the INS-1 rat insulinoma cell line, a well-validated *in vitro* model^28^. We investigated glucose-induced insulin secretion in INS-1 cells that had been chronically pre-exposed for 24 or 48 h to atorvastatin or pravastatin (10 or 100 ng/mL). Under control conditions, insulin concentrations in the medium rose from 32.3 ± 3.5 ng/mg of protein/h at 2.8 mM of glucose to 93.4 ± 7.9 ng/mg of protein/h at 22.2 mM of glucose (fold-change of 2.9 ± 0.4, p < 0.001). Pre-incubation with atorvastatin impaired both basal and glucose-stimulated insulin release in a time and dose-dependent manner with a maximal effect observed after 48 h exposure to atorvastatin 100 ng/mL (Fig. 2, Panel A).

As seen in pancreatic islets, both basal and glucose-induced insulin release from INS-1 pre-exposed to pravastatin was similar to control cells. (Fig. 2, Panel B).

### Atorvastatin but not pravastatin reduced ATP production in INS-1 cells

**Figure 3:**
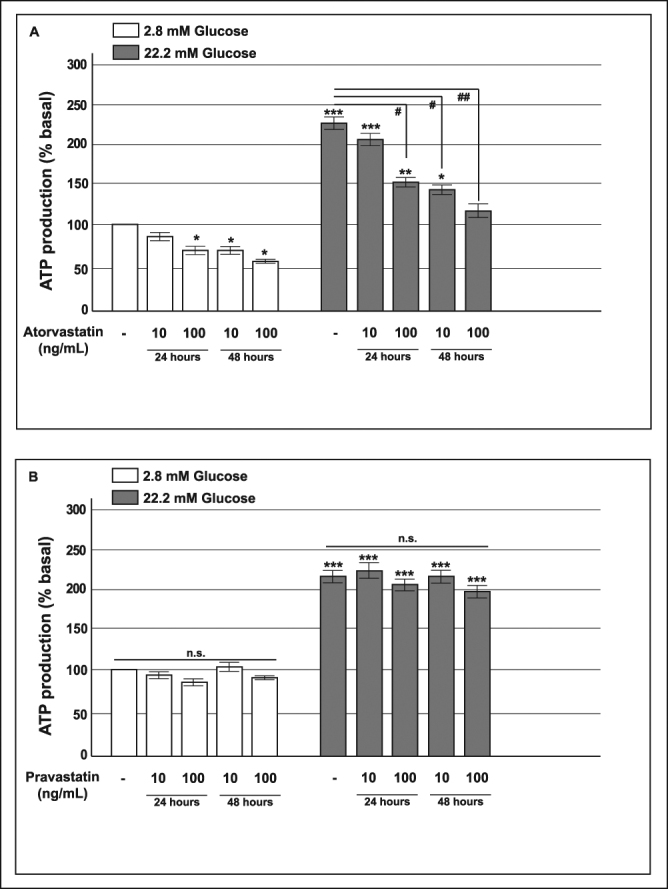
Effect of atorvastatin and pravastatin on glucose-induced ATP synthesis in INS-1 cells. Panel A: acute glucose-induced ATP production in control cells and in cells pre-exposed to 10 or 100 ng/mL of atorvastatin for 24 or 48 h; Panel B: acute glucose-induced ATP production in INS-1 cells pre-exposed to 10 or 100 ng/mL of pravastatin for 24 or 48 h. *P < 0.05, **P < 0.01, ***P < 0.001 vs. control at 2.8 mM glucose; ^#^P < 0.05, ^##^P < 0.01 vs. control at 22.2 mM glucose; n.s. not significant (1-way ANOVA followed by Bonferroni test, n = 4).

Because the rise of ATP plays a pivotal role in glucose-induced insulin release by causing K^+^-ATP channel closure, membrane depolarization, increased calcium influx, and insulin granule exocytosis, we measured ATP levels in INS-1 cells that were chronically exposed to pravastatin or atorvastatin (Fig. 3).

In control cells, 22.2 mM glucose acutely stimulated ATP production (fold-changes of 2.2 ± 0.4, p < 0.001) compared with the ATP production levels observed in the presence of 2.8 mM glucose.

In cells that had been pre-exposed to atorvastatin, basal (2.8 mM glucose) ATP levels were lower than those in control cells; furthermore, glucose-stimulated (22.2 mM glucose) ATP production was markedly reduced in a dose-time dependent manner (Fig. 3, Panel A).

In contrast, in INS-1 cells that had been pre-exposed to pravastatin both basal and glucose-induced ATP production were unaffected (Fig. 3, Panel B) by treatment. These data indicated that chronic exposure to atorvastatin affected glucose-induced insulin secretion through direct actions on mitochondrial ATP production.

### Atorvastatin reduced mitochondrial OxPhos complexes expression in INS-1 cells

Mitochondrial metabolism generates more than 90% of the ATP required for cellular processes^29^. ATP synthesis depends on the transfer of electrons through the respiratory electron transport chain complexes I–IV in the inner mitochondria membrane and on the consequent phosphorylation of ADP into ATP by the F_1_ ATP-synthase (Complex V).

**Figure 4:**
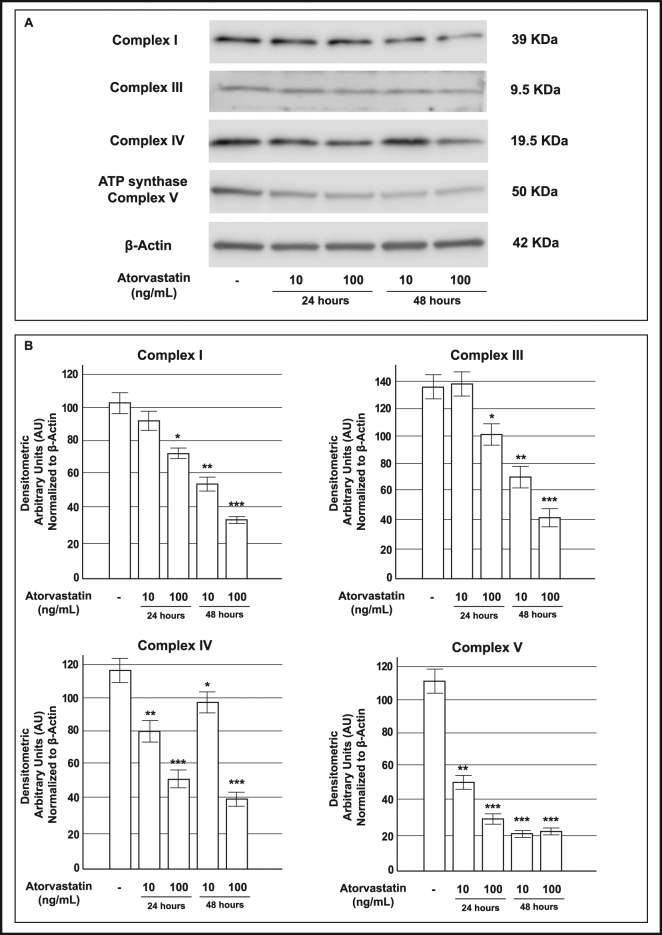
Effect of pre-exposure to atorvastatin on protein expression of mitochondrial respiratory chain complexes in INS-1 cells. Panel A: representative Western blots for: (from the top to bottom) the 39 KDa subunit (alpha subcomplex, 9) of the NADH-ubiquinone oxidoreductase (Complex I), the 10 KDa subunit (subunit VII) of the Ubiquinone-cytochrome *c* (Complex III), the 19.6 KDa subunit IV (COX IV) of the Cytochrome *c* oxidase (Complex IV), the 56.6 KDa β subunit (F_1_ complex, beta subunit) of the ATP synthase (Complex V) and Actin in control cells and in cells that had been exposed to atorvastatin 10 or 100 ng/mL for 24 or 48 h. The cropped blots were run under the same experimental conditions and β-Actin signal was used to normalize the data. The full-length blots are presented in Supplementary Figure 1. Panel B: corresponding densitometric analysis. *P < 0.05, **P < 0.01, ***P < 0.001 vs. control (1-way ANOVA followed by Bonferroni test, n = 3).

On these bases, to elucidate the mechanism by which atorvastatin reduced ATP levels, we studied mitochondrial respiratory chain complexes expression in INS-1 cells chronically (24 or 48 h) treated with atorvastatin (10 or 100 ng/mL) (Fig. 4). In particular, we analyzed, by Western blot, protein levels of representative subunit of the complexes I (NADH-ubiquinone oxidoreductase), III (ubiquinol-cytochrome *c* reductase), IV (cytochrome c oxidase) and V (F_0_F_1_-complex).

**Figure 5:**
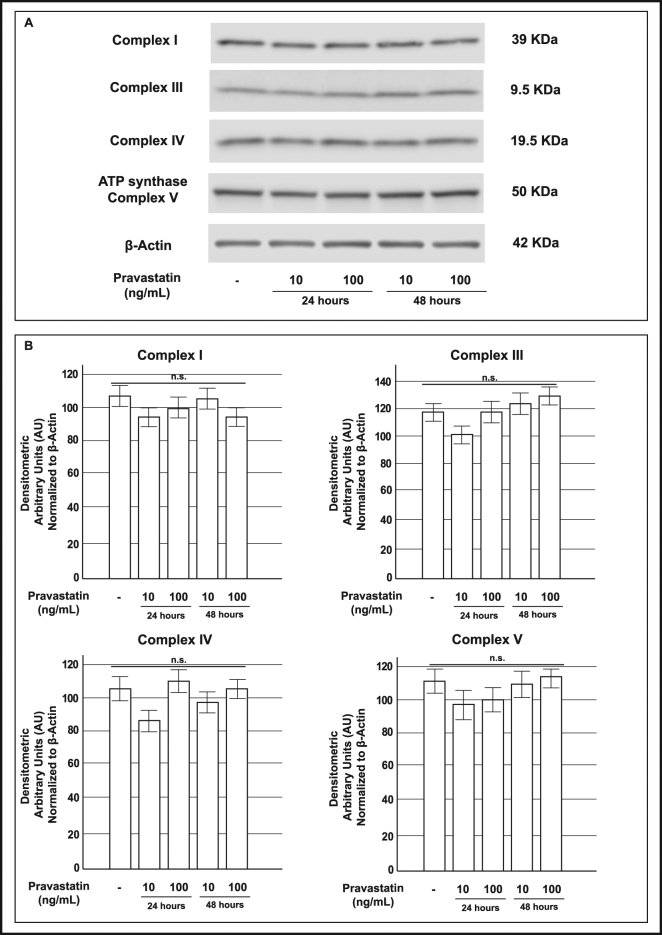
Effect of pre-exposure to pravastatin on protein expression of mitochondrial respiratory chain complexes in INS-1 cells. Panel A: representative Western blots for: (from the top to bottom) the 39 KDa subunit (alpha subcomplex, 9) of the NADH-ubiquinone oxidoreductase (Complex I), the 10 KDa subunit (subunit VII) of the Ubiquinone-cytochrome *c* (Complex III), the 19.6 KDa subunit IV (COX IV) of the Cytochrome *c* oxidase (Complex IV), the 56.6 KDa β subunit (F_1_ complex, beta subunit) of the ATP synthase (Complex V) and Actin in control cells and in cells that had been exposed to pravastatin 10 or 100 ng/mL for 24 or 48 h. The cropped blots were run under the same experimental conditions and β-Actin signal was used to normalize the data. The full-length blots are presented in Supplementary Figure 1. Panel B: corresponding densitometric analysis. n.s. not significant (1-way ANOVA followed by Bonferroni test, n = 3).

We found that complex protein levels were significantly decreased in cells that had been pre-exposed to atorvastatin compared to control cells with a maximal effect at the highest dose-time combination (100 ng/mL for 48 h) (Fig. 4, Panel B). In contrast, in INS-1 cells that had been pre-exposed to pravastatin there were no significant differences in the abundance of these proteins between the control and treated groups (Fig. 5).

### Atorvastatin increased ROS production in INS-1 cells

Statins have been shown to increase intracellular reactive oxygen species in different models^22, 23, 30^. To investigate if the observed mitochondrial defects could arise from increased oxidative stress, we measured oxygen free radical production in cells cultured for 24 or 48 h with atorvastatin (10 or 100 ng/mL).

**Figure 6:**
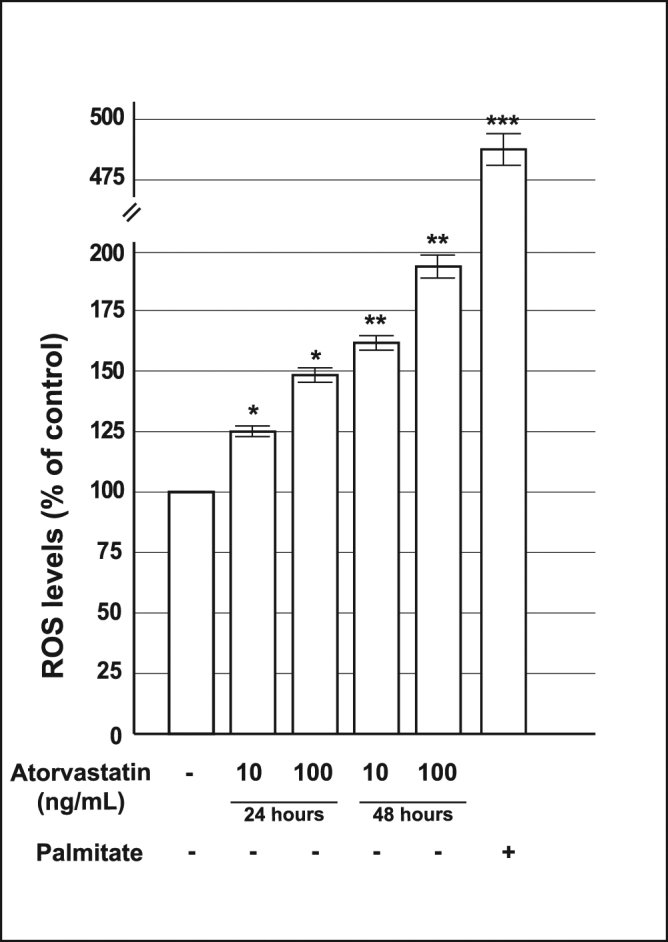
Effect of pre-exposure to atorvastatin on reactive oxygen species (ROS) production in INS-1 cells. ROS production in control cells and cells pre-exposed to 10 or 100 ng/mL of atorvastatin for 24 or 48 h; palmitate 0.25 mM was used as a positive control. *P < 0.05, **P < 0.01, ***P < 0.001 vs. control (1-way ANOVA followed by Bonferroni test, n = 3).

As shown in Fig. 6, chronic exposure to atorvastatin produced a significant increase of ROS levels in a dose-time dependent manner; indicating that chronic atorvastatin treatment is detrimental for pancreatic beta cell function as a result of the enhanced oxidative stress.

### Mevalonate rescued the mitochondrial and secretory defects caused by atorvastatin

Statins act by competitively inhibiting HMG-CoA reductase activity and blocking the conversion of HMG-CoA to mevalonate. The synthesis of mevalonate is upstream in a sequence of reactions, collectively known as the mevalonate pathway; this constitutes a complex biochemical pathway required for the generation of several fundamental end-products including sterols, isoprenoids, dolichol, ubiquinone, and isopentenyladenine^31^.

**Figure 7:**
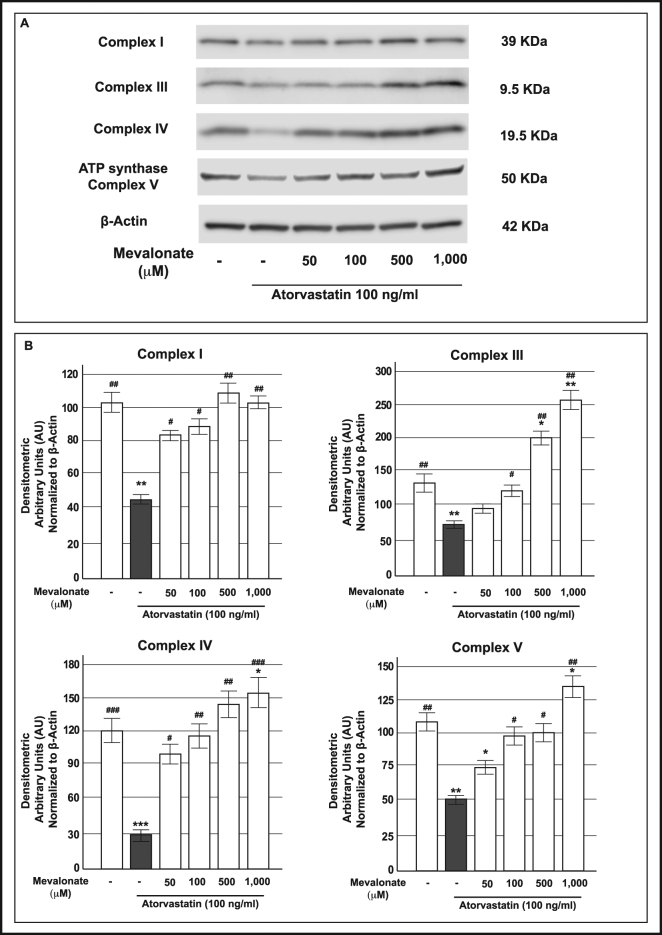
Effect of mevalonate addition, in INS-1 cells simultaneously exposed to atorvastatin, on protein expression of mitochondrial respiratory chain complexes. Panel A: representative Western blots for: (from the top to bottom) the 39 KDa subunit (alpha subcomplex, 9) of the NADH-ubiquinone oxidoreductase (Complex I), the 10 KDa subunit (subunit VII) of the Ubiquinone-cytochrome *c* (Complex III), the 19.6 KDa subunit IV (COX IV) of the Cytochrome *c* oxidase (Complex IV), the 56.6 KDa β subunit (F_1_ complex, beta subunit) of the ATP synthase (Complex V) and Actin in control cells and in cells that had been in parallel exposed to atorvastatin 100 ng/mL and mevalonate at the reported concentrations for 48 h. The cropped blots were run under the same experimental conditions and β-Actin signal was used to normalize the data. The full-length blots are presented in Supplementary Figure 1. Panel B: corresponding densitometric analysis. *P < 0.05, **P < 0.01, ***P < 0.001 vs. control; ^#^P < 0.05, ^##^P < 0.01, ^###^P < 0.001 vs 100 ng/mL atorvastatin alone treated cells (gray bars) (1-way ANOVA followed by Bonferroni test, n = 3).

In order to determine whether the impairments induced by atorvastatin resulted from the inhibition of the mevalonate pathway, we added increasing concentrations of mevalonate (50, 100, 500 or 1000 µM) to INS-1 cells simultaneously treated with 100 ng/mL atorvastatin for 48 h. As evidenced by Western Blot analysis, co-incubation with mevalonate prevented atorvastatin-induced reduction of the mitochondrial complexes with a statistically significant effect for all the proteins at the concentration of 500 µM (Fig. 7).

**Figure 8:**
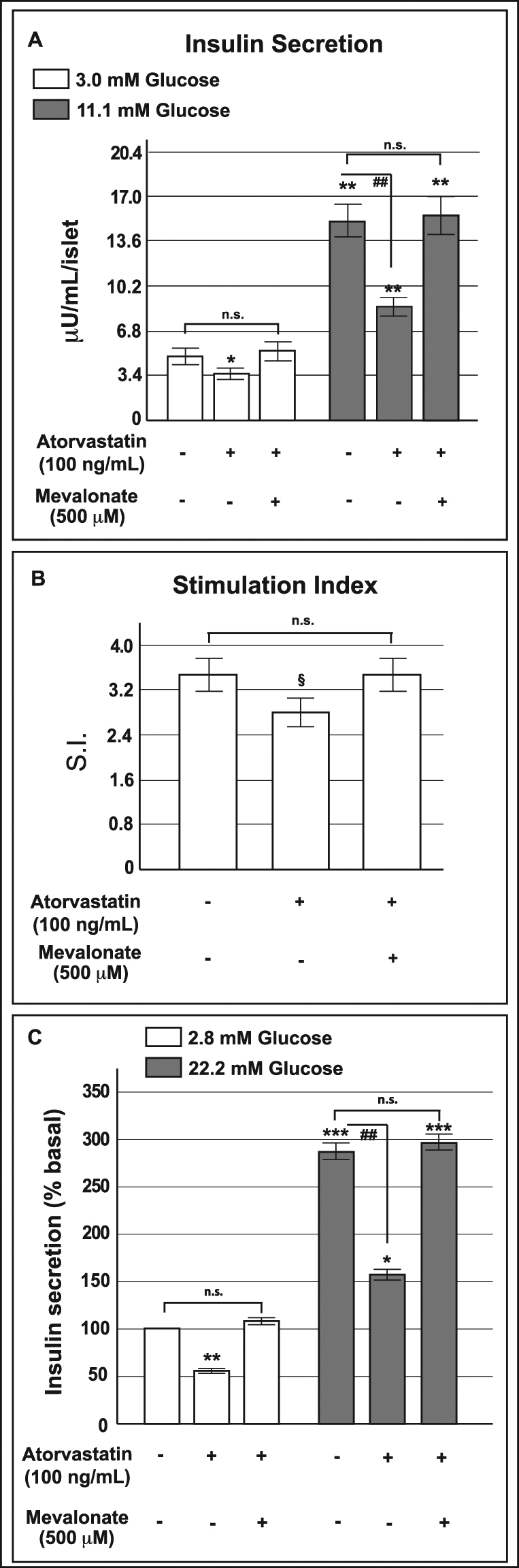
Effect of mevalonate addition in human pancreatic islets and INS-1 cells simultaneously exposed to atorvastatin, on glucose-induced insulin release. Absolute glucose-induced insulin secretion (expressed as µU/mL/islet) (Panel A) and relative stimulation index (S.I.) (Panel B) in control human pancreatic islets and in islets in parallel pre-exposed to atorvastatin 100 ng/mL and mevalonate 500 μM for 48. *P < 0.05, **P < 0.01 vs. control at 3.0 mM glucose; ^##^P < 0.01 vs. control at 11.1 mM glucose; ^§^P < 0.05 vs S.I. in control islets; n.s. not significant (1-way ANOVA followed by Bonferroni test, n = 5); Panel C: acute glucose-induced insulin secretion in control cells and in cells in parallel pre-exposed to atorvastatin 100 ng/mL and mevalonate 500 μM for 48 h (baseline secretory rate at 2.8 mM glucose: 45.4 ± 5.1 ng/mg of protein in 1 h). *P < 0.05, **P < 0.01, ***P < 0.001 vs. control at 2.8 mM glucose; ^##^P < 0.01 vs. control at 22.2 mM glucose; n.s. not significant (1-way ANOVA followed by Bonferroni test, n = 4).

On the basis of these findings, we decided to evaluate the effect of the addition of 500 µM mevalonate to both human pancreatic islets and INS-1 cells treated at the same time with atorvastatin (100 ng/mL for 48 h) on acute glucose stimulated insulin secretion (Fig. 8). As shown in Panel A of Fig. 8, in islets pre-exposed to atorvastatin 100 ng/mL and co-incubated with 500 µM mevalonate a clear reversal of the insulin release pattern to control conditions was observed; as a consequence, the addition of mevalonate induced a significant (p < 0.05) increase in the IS value compared to islets treated with atorvastatin 100 ng/mL alone (Fig. 8, Panel B). Similarly, the secretory defects induced by atorvastatin in INS-1 cells were completely prevented by the contemporary presence of mevalonate (Fig. 8, Panel C), indicating that glucose-induced insulin secretion is altered by atorvastatin through the direct effects of the drug on the mevalonate pathway.

### Atorvastatin reduced COQB10 expression and mevalonate reversed the defect in INS-1 cells

Mevalonate also constitutes a precursor of Coenzyme Q10 or ubiquinone, consequently the action of statins on the mevalonate pathway decreases levels of coenzyme Q10, which is considered an important antioxidant defense system.

**Figure 9:**
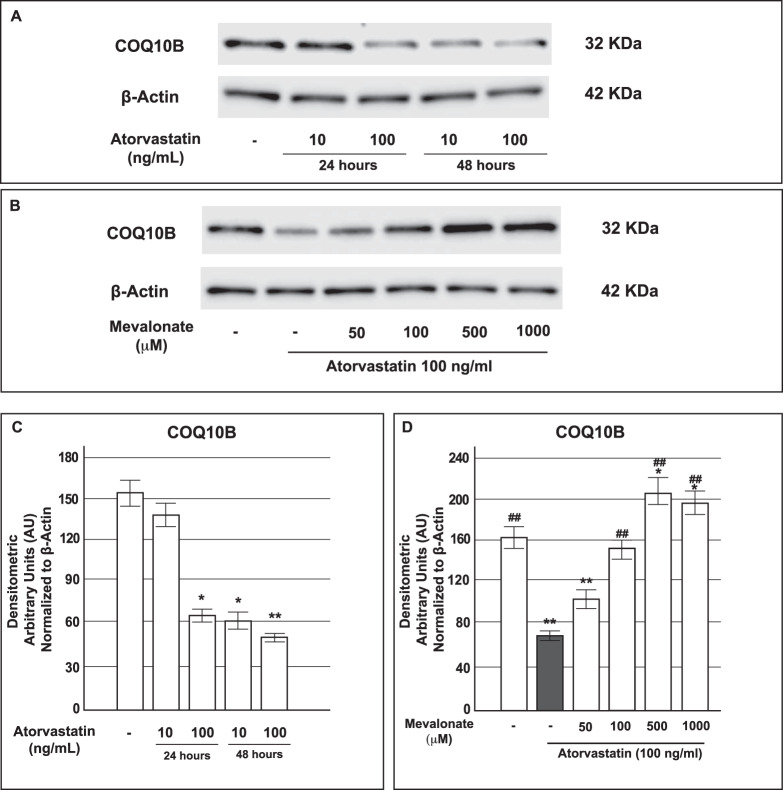
Effect of exposure to atorvastatin and co-treatment with mevalonate on protein expression of COQ10B in INS-1 cells. Panel A: representative Western blots for COQ10B and Actin in control cells and in cells that had been in parallel exposed to atorvastatin 10 or 100 ng/mL of atorvastatin for 24 or 48 h. The cropped blots were run under the same experimental conditions. The full-length blots are presented in Supplementary Figure 1; Panel C: corresponding densitometric analysis; Panel B: representative Western blots for COQ10B and Actin in control cells and in cells that had been in parallel exposed to atorvastatin 100 ng/mL and mevalonate at the reported concentrations for 48 h. The cropped blots were run under the same experimental conditions and β-Actin signal was used to normalize the data. The full-length blots are presented in Supplementary Figure 1. Panel D: corresponding densitometric analysis. *P < 0.05, **P < 0.01 vs. control; ^##^P < 0.001 vs 100 ng/mL atorvastatin alone treated cells (gray bar) (1-way ANOVA followed by Bonferroni test, n = 3).

In order to elucidate if increased oxidative stress and related disorders were due to a reduced expression of coenzyme Q10, we also measured the levels of COQ10 protein, that functions in the correct activity of CoQ10 as electron carrier in the mitochondrial electron transport chain. In particular, we measured COQ10B protein expression in INS-1 cells chronically (24 or 48 h) treated with atorvastatin (10 or 100 ng/mL). As shown in the Panels A and C of Fig. 9, our results showed a significant decrease in COQ10B expression in atorvastatin-treated cells compared with control cells, as evaluated by Western Blot analysis.

On the basis of this evidence, and in order to determine whether this reduction primarily resulted from the inhibition of the mevalonate pathway, we studied the expression of COQ10B protein after the addition of increasing concentrations of mevalonate (50, 100, 500 or 1000 μM) to INS-1 cells contemporarily treated with 100 ng/mL atorvastatin for 48 h. As evidenced by Western Blot analysis, mevalonate increased the expression of COQ10B with a statistically significant effect at the concentration of 500 μM (Fig. 9, Panel B and D).

### N-AcetylCysteine protected mitochondrial respiration and insulin release from deleterious effects of atorvastatin in INS-1 cells

**Figure 10:**
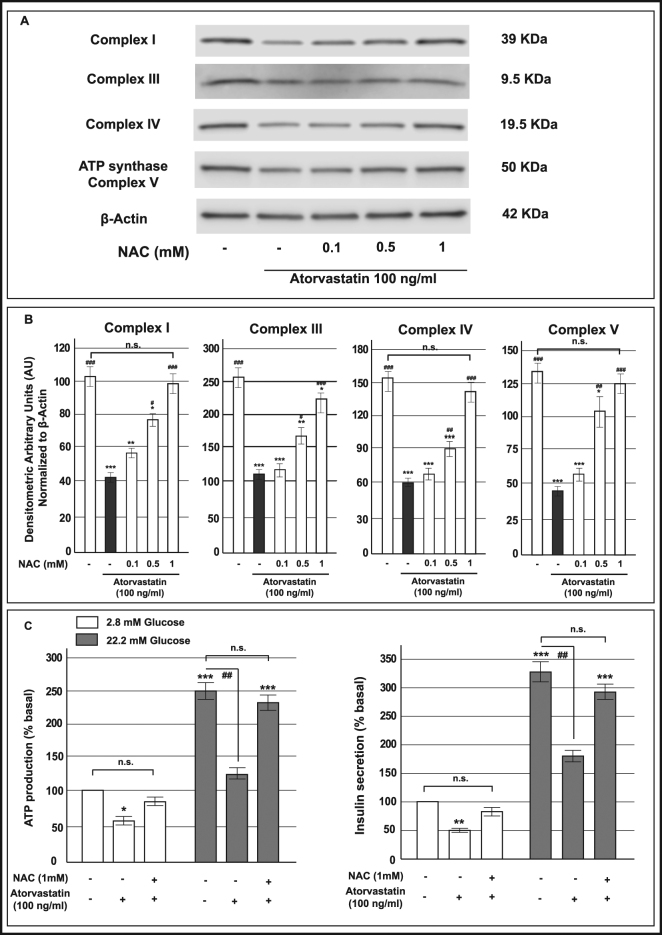
Effect of exposure to atorvastatin and co-treatment with NAC on protein expression of mitochondrial respiratory chain complexes, glucose-induced insulin release and ATP synthesis in INS-1 cells. Panel A: representative Western blots for: (from the top to bottom) the 39 KDa subunit (alpha subcomplex, 9) of the NADH-ubiquinone oxidoreductase (Complex I), the 10 KDa subunit (subunit VII) of the Ubiquinone-cytochrome *c* (Complex III), the 19.6 KDa subunit IV (COX IV) of the Cytochrome *c* oxidase (Complex IV), the 56.6 KDa β subunit (F_1_ complex, beta subunit) of the ATP synthase (Complex V) and Actin in control cells and in cells that had been in parallel exposed to atorvastatin 100 ng/mL and NAC (0.1, 0.5 and 1 mM) for 48 h. The cropped blots were run under the same experimental conditions and β-Actin signal was used to normalize the data. The full-length blots are presented in Supplementary Figure 1. Panel B: corresponding densitometric analysis. *P < 0.05, **P < 0.01, ***P < 0.001 vs. control; ^#^P < 0.05, ^##^P < 0.01, ^###^P < 0.001 vs 100 ng/mL atorvastatin alone treated cells (gray bars) (1-way ANOVA followed by Bonferroni test, n = 3). Panel C (left-side): acute glucose-induced ATP production in control cells and in cells in parallel pre-exposed to atorvastatin 100 ng/mL and NAC 1 mM for 48 h. Panel C (right-side): acute glucose-induced insulin secretion in control cells and in cells in parallel pre-exposed to atorvastatin 100 ng/mL and NAC 1 mM for 48 h (baseline secretory rate at 2.8 mM glucose: 32.4 ± 3.7 ng/mg of protein in 1 h). *P < 0.05, **P < 0.01, ***P < 0.001 vs. control at 2.8 mM glucose; ^##^P < 0.01 vs. control at 22.2 mM glucose; n.s. not significant (1-way ANOVA followed by Bonferroni test, n = 4).

To investigate the mechanisms behind the beneficial effects of mevalonate in atorvastatin-treated cells, we evaluated the effect of a co-treatment with the antioxidant N-AcetylCysteine (NAC) in our system. We started to analyze mitochondrial complexes expression in INS-1 cells treated with increasing concentration of NAC (0.1, 0.5 and 1 mM) and co-cultured in the presence of atorvastatin 100 ng/mL for 48 h. As evidenced by Western Blot analysis, co-incubation with NAC rescued atorvastatin-induced reduction of the mitochondrial complexes with a statistically significant effect for all the proteins at the concentration of 1 mM (Fig. 10, Panels A and B). On the basis of these data we evaluated the effect of the addition of NAC 1 mM to INS-1 cells co-cultured with atorvastatin, on glucose-induced ATP production and insulin-secretion. As shown in the Panel C of the Fig. 10, we found that NAC 1 mM, by augmenting the electron transport chain proteins, prevented the reduction of ATP synthesis and consequently, rescued the secretory defects induced by atorvastatin; thus, indicating that both mitochondrial respiration and insulin secretion are impaired by atorvastatin through the increase of oxidative stress.

## Discussion

Statins are one of the most highly prescribed classes of drugs to prevent CV diseases, the leading cause of death worldwide^32, 33^. Although evidently lifesaving, recent clinical data have shown that chronic statin therapy is associated with an increased risk of type 2 diabetes mellitus (T2DM)^5, 6, 8, 34^, however, the molecular mechanisms behind this association are not yet fully understood.

In this work, we provide evidence that chronic exposure to atorvastatin (lipophilic statin), but not to pravastatin (hydrophilic statin), inhibited both basal and glucose-stimulated insulin secretion in pancreatic human islets and in INS-1 β-cells. In addition, we demonstrate for the first time that this secretory alteration is associated with mitochondrial dysfunctions induced by conditions of oxidative stress.

Our data confirm the observations that the detrimental effects of HMG-CoA reductase inhibitors are dose and potency dependent and strictly related to their lipophilicity^6, 10, 27, 35, 36^. Accordingly, previous studies reported that lipophilic statins, such as atorvastatin, have negative effects on pancreatic β-cell function, while for hydrophilic statins, such as pravastatin, neutral or improving effects have been observed^27, 37, 38^.

Furthermore, we observed that while pravastatin affected neither insulin secretion nor mitochondrial metabolism, atorvastatin not only impaired insulin release and glucose metabolism but also increased ROS production and suppressed CoQ10, one of the major cellular defense systems to oxidative stress.

These data provided a new molecular mechanism behind the dysregulation of β-cell secondary to statin exposure. Previous studies addressing this topic have shown other potential molecular mechanisms, including reduction of GLUT2 expression^39^, impairment of G protein action^40^ and inhibition of L-type calcium channels^27^, key elements for insulin secretion. The pivotal point of our results is, instead, the mitochondria; in pancreatic β-cells, with low antioxidant capacities, atorvastatin decreased COQ10B protein levels and induced high oxidative stress, responsible for mitochondrial deterioration. Previous reports have already evidenced the association between statins and ROS in other models. Simvastatin has been reported to significantly increase mitochondrial oxidative stress and reduce ATP synthesis both in human hepatocytes (HepG2) and in primary human myotubes^41, 42^. Moreover, treatment with atorvastatin has been shown to be associated with mitochondrial oxidative stress in deltoid biopsies of patients with statin-associated myopathy^23^.

Bouitbir *et al*., investigating the mechanisms by which statins exert their beneficial and detrimental effects, found opposite effects of these drugs on skeletal and cardiac muscles. In particular, treatment with atorvastatin has been observed to be associated with increased ROS production in both types of muscle but in cardiac muscle, with high anti-oxidative capacity, this translates into activation of mitochondrial biogenesis pathways and improvement of metabolic health; conversely, in skeletal muscle, where ROS-detoxifying constituents are weak, this induced mitochondrial dysfunctions, down-regulation of mitochondrial biogenesis and myopathy^22^.

Similarly to skeletal muscle, insulin secreting, β-cells are particularly susceptible to oxidative damage because of the low expression of natural enzymatic defenses, i.e. catalase and superoxide dismutase^43, 44^. Accordingly, in our model the increased oxidative stress, following atorvastatin treatment, impaired mitochondrial function and blunted insulin secretion.

Additionally, we found a reduction of mitochondrial complexes and a consequent impairment of ATP production in INS-1 cells chronically treated with atorvastatin. Indeed, one of the primary targets of ROS are not only the mitochondrial DNA but also the proteins of the inner mitochondrial membrane, which undergo an extensive oxidation in conditions of mitochondrial oxidative stress^45^.

Our data also evidenced that chronic exposure to atorvastatin reduced the expression of COQ10B protein in pancreatic β-cell. Coenzyme Q10 is a product of the mevalonate pathway and in addition to constituting an important electron transporter of the mitochondrial respiratory chain, it is the only lipid-soluble antioxidant normally synthesized by the organism^46^. The role of CoQ10B protein depletion in our system is critical by virtue of the low anti-oxidative capacities of β-cells. Consequently, the potential reduction of CoQ10 functional activity, in a model where ROS-detoxifying constituents are already low, leads to increased generation of ROS which ultimately results in deterioration of the mitochondrial respiratory function^47^.

Previous studies have already reported that suppression of CoQ10 by statins increased oxidative stress affecting mitochondria in skeletal muscle^20, 22, 23^. In HepG2 cells, it has been shown that treatment with simvastatin decreased CoQ10 levels and increased oxidative damage reducing ATP synthesis; in the same model the supplementation of CoQ10 reduced oxidative stress and reversed ATP synthesis^42^. Moreover, human studies have reported reduced levels of CoQ10 in hypercholesterolemic patients in treatment with atorvastatin^48–50^ or simvastatin^20^.

Interestingly, our data indicated that the detrimental effects of atorvastatin on pancreatic β-cells were reversed by co-treatment with mevalonate. These observations indicate that the ability of statins in increasing T2DM is not due to off-target effects of the drug but rather to side effects of its effective blockage of HMG-CoA reductase; therefore, lacking downstream products of the mevalonate pathway must be responsible for the observed effects. Likewise, genetic variants that reduce the activity of HMG-CoA reductase (which is analogous to inhibiting this enzyme with a statin) have been associated with an increased incidence of diabetes^51^.

In our study, mevalonate co-treatment allowed replenishment of CoQ10 and oxphos complexes, preventing statin-induced β-cell dysfunction. Similarly in cells cultured with atorvastatin, the contemporary addition of the antioxidant NAC was also able to abolish statin-induced β-cell defects. Together, these data demonstrated the existence of a link between statin-secondary mevalonate depletion, ROS accumulation and β-cell dysfunction, suggesting that the potentially decreased functional activity of CoQ10 and the consequent high oxidative stress, in a limited ROS-scavenging system, could be the triggering factor inducing mitochondrial dysfunction and down-regulation of insulin secretion.

These interesting findings could provide support for the efficacy of co-administering CoQ10 with statins in order to prevent or reduce the risk of statin-induced diabetes.

The benefits of CoQ10 supplementation to reduce the incidence of statin side effects have been widely shown in other models. Recently, Muraki *et al*. have demonstrated that co-administration of CoQ10 reversed mitochondrial dysfunction and exercise intolerance in mice with ubiquinone deficiency induced by atorvastatin^52^. Furthermore, it has been observed that the increased insulin-resistance in adipocytes following treatment with simvastatin can be prevented by co-incubation with CoQ10^53^. Clinical studies of ubiquinone supplementation have evidenced the effectiveness in ameliorating neurodegenerative diseases, heart failure, and muscular symptoms during therapies with statins^54–56^.

In conclusion, the present study supports evidences regarding the different diabetogenicity of lipophilic and hydrophilic statins and demonstrates, for the first time, a direct deleterious effect of these drugs on mitochondria due to the suppression of the antioxidant defense system and induction of ROS production in a model of human pancreatic β-cells.

This may have important clinical implications because it indicates the existence of a novel mechanism linking treatment with lipophilic statins to the increased risk of type 2 diabetes. It also suggests that coenzyme Q may be a potential target for preserving β-cell function during statin-therapy. Finally, these data may help to design strategies for prevention or reduction of statin induced β-cell dysfunction and diabetes in patients treated with lipophilic statins.

## Materials and Methods

### Antibodies and Reagents

Antibodies were purchased from Thermo Fisher Scientific (Cambridge, UK), Santa Cruz Biotechnology (Santa Cruz, CA, USA), Abcam (Cambridge, UK), GE Healthcare Life Sciences (Little Chalfont, UK) and Sigma-Aldrich (St. Louis, MO, USA). Fetal bovine serum (FBS) was provided by Thermo Fisher Scientific (Cambridge, UK). Cell media, statins, mevalonate, palmitate, 2′,7′-Dichlorofluorescein diacetate, N-AcetylCysteine (NAC) and all v, MO, USA), unless otherwise stated.

### Pancreatic islets isolation and INS-1 cell culture

Islets were prepared from nine multi-organ non-diabetic donors by collagenase digestion followed by density gradient purification^57^. Human pancreata were harvested from brain-dead organ donors after informed consent was obtained in writing from family members, and processed for islet isolation, if not suitable for clinical whole organ purposes, according the procedures approved by the ethics committee of the University of Pisa.

After isolation, the islets were maintained for 2–3 days in M199 medium, containing 5.5 mmol/l glucose, supplemented with 10% serum and antibiotics. Then, batches of approximately 1,000 islets were cultured for 48 h in M199 medium either with or without the addition of 10 or 100 ng/mL atorvastatin or pravastatin.

INS-1 cells were cultured in RPMI 1640 medium containing 11 mM glucose, 10 mM HEPES, 10% heat-inactivated FBS, 2 mM glutamine, 1 mM sodium pyruvate, 50 µM β-mercaptoethanol, 100 IU/mL penicillin and 100 µg/mL streptomycin in a humidified atmosphere (5% CO_2_ −95% air).

The medium was changed once a week, and the cells were trypsinized and reseeded at a 1:3 dilution when 70% confluence was reached (approximately every 5 days). Most of the experiments were conducted with cells at passages 12–22; however, the cellular responses were similar for passages 10–25.

Twenty-four hours after plating, INS-1 cells were cultured for 24 or 48 h in complete RPMI 1640 medium in the presence or absence of 10 or 100 ng/mL atorvastatin or pravastatin. In specific sets of experiments, the cells were also incubated with mevalonate or N-AcetylCysteine (NAC) for 48 h prior to experiments. At the end of each experiment, the cells were lysed with RIPA (Radio-ImmunoPrecipitation Assay) buffer and the lysates were analyzed for total protein content, determined with the BCA assay (Thermo Fisher Scientific, Cambridge, UK). To select the appropriate normalization method, we performed preliminary experiments and measured the cell number and total protein content in wells at the end of the culture period. No differences in normalized data were observed between the two normalization methods. On the basis of these findings, we chose to normalize all of the measurements to the total protein content per well.

### Insulin release

For insulin secretion studies, batches of 15 islets were first kept at 37 °C for 45 min in Krebs–Ringer Bicarbonate Solution (KRB), 0.5% (vol./vol.) albumin, pH 7.4, containing 3.0 mmol/l glucose (wash-out phase). Then, the medium was replaced with KRB containing 3.0 mmol/l glucose to assess basal insulin secretion (45 min), followed by a further 45 min incubation with 11.1 mmol/l glucose to assess insulin response to acute challenge.

INS-1 cells were plated into 48-well plates (15 × 10^4^ cells/well) and cultured as described above. At the end of the culture period, cells were washed with phosphate-buffered saline (PBS) and pre-incubated for 30 min at 37 °C in 0.5 mL of Krebs Ringer Hepes (KRH) buffer (containing 134 mM NaCl, 3.6 mM KCl, 0.5 mM NaH_2_ PO_4_, 0.5 mM MgCl_2_, 1.5 mM CaCl_2_, 10 mM HEPES, 2.8 mM glucose, 0.1% BSA, pH 7.40). Cells were then incubated for 1 h at 37 °C in 0.5 mL of fresh KRH buffer containing 2.8 or 22.2 mM glucose.

At the end of the incubation period, the media were collected and centrifuged to remove any floating cells. Insulin was measured in the supernatants using an enzyme-linked immunosorbent assay (Millipore Co., Billerica, MA). Each sample was analyzed in duplicate. Insulin secretion was normalized to the baseline levels in 2.8 mM glucose, which were measured in parallel on the same day.

### Measurement of intracellular ATP content in INS-1 cells

ATP levels were measured with the CellTiter-Glo Luminescent Cell Viability Assay (Promega, Madison, WI, USA) as previously described^58^. Briefly, equal numbers of cells were cultured for 24 or 48 h at 37 °C in complete medium in the presence or absence of 10 or 100 ng/mL atorvastatin or pravastatin. Then, the cells were washed and acutely exposed to different glucose concentrations (2.8 or 22.2 mM).

The reaction agent was added to each well, and the plate was incubated at room temperature for 10 min. The luminescent signal was proportional to the amount of ATP. The ATP levels were normalized to the baseline levels in 2.8 mM glucose, which were measured in parallel on the same day.

### Western blot analysis in INS-1 cells

Proteins were extracted from INS-1 cells with RIPA lysis buffer, and Western blotting was performed as previously described^59^. Membranes were incubated with primary antibodies (used at a final dilution 1:1000 in 5% BSA, over night at 4 °C with gentle agitation) for representative subunit of the Complexes I, IV (Thermo Fisher Scientific, Cambridge, UK), III, V (Santa Cruz Biotechnology, Santa Cruz, CA, USA) and for Coenzyme Q10 (Abcam, Cambridge, UK) and then exposed to anti-rabbit, anti-mouse (GE Healthcare Life Sciences, Little Chalfont, UK) or anti-goat (Santa Cruz Biotechnology) secondary antibodies (used at a final dilution 1:2000 in 5% milk, 1 hour at room temperature with gentle agitation), conjugated to horseradish peroxidase. In every experiment, we normalized data and checked that an equal amount of protein was loaded by reprobed the nitrocellulose membranes with a β-Actin (Sigma-Aldrich, St. Louis, MO, USA) monoclonal antibody (used at a final dilution 1:10000). All of the immunoblot signals were visualized using the Odyssey Fc System infra-red scanner (LI-COR Biosciences, Lincoln, NE, USA) and were subjected to densitometric analyses using Odyssey software Image studio Lite Ver 5.2 as previously reported^60^.

### ROS measurement in INS-1 cells

Accumulation of intracellular ROS was estimated by fluorescence assay using 2,7′-Dichlorofluorescein diacetate (H_2_DCF-DA; Sigma-Aldrich) as a probe. H_2_DCF-DA readily diffuses into the cells where it is enzymatically hydrolyzed by intracellular esterase to H_2_DCF and subsequently oxidized to the highly fluorescent compound DCF in the presence of ROS. The intensity of fluorescence is proportional to the intracellular levels of ROS.

After atorvastatin treatments cells were incubated with pre-warmed basal medium containing 10 µM H_2_DCF-DA for 30 min at 37 °C. Stained cells were gently rinsed in basal medium and incubated again for 1 h with medium containing atorvastatin (10 or 100 ng/mL). The cells were washed twice with PBS and fluorescence was monitored in a microplate fluorometer (Victor^2^; Perkin-Elmer, Singapore) at 495 nm excitation and 530 nm emission. All the obtained values were normalized on cell proliferation by Crystal Violet assay. In a specific set of experiments, cells were incubated with palmitate 0.25 mM for 48 h; this fatty acid is known to stimulate oxidative activity in β-cells^61^ and was used as a positive control.

### Statistical analysis

The differences between the means of unpaired samples were analyzed using Student’s t test. Comparisons between multiple means were performed via ANOVA followed by *post hoc* analysis for significance (Bonferroni test). For both tests, the level of significance was set at p < 0.05. The statistical analyses were performed using GraphPad Prism 5.0 (GraphPad Software, Inc., San Diego, CA, USA). The data are expressed as the means ± SEM.

### Data Availability Statement

All relevant data are within the paper and its Supporting Information file.

## Electronic supplementary material

Supplementary Information
