# Author Correction: Atorvastatin but Not Pravastatin Impairs Mitochondrial Function in Human Pancreatic Islets and Rat β-Cells. Direct Effect of Oxidative Stress

**DOI:** 10.1038/s41598-020-78915-w

**Published:** 2020-12-10

**Authors:** Francesca Urbano, Marco Bugliani, Agnese Filippello, Alessandra Scamporrino, Stefania Di Mauro, Antonino Di Pino, Roberto Scicali, Davide Noto, Agata Maria Rabuazzo, Maurizio Averna, Piero Marchetti, Francesco Purrello, Salvatore Piro

**Affiliations:** 1 Department of Clinical and Experimental Medicine, Garibaldi Hospital, University of Catania, Catania, Italy; 2 Department of Clinical and Experimental Medicine, Islet Cell Laboratory, University of Pisa, Pisa, Italy; 3 Department of Biomedicine, Internal Medicine and Medical Specialties (DIBIMIS), University of Palermo, Palermo, Italy

Correction to: *Scientific Reports* 10.1038/s41598-017-11070-x, published online 19 September 2017

The original version of this Article contained errors.

In the original version of this Article, the authors inaccurately reported that they had measured expression of coenzyme Q10. The antibody used in the study detects COQ10B, a mitochondrial protein which interacts with coenzyme Q10 during its correct function. The description of this experiment and its results are now corrected to reflect this.

In the Results, the heading,

“Atorvastatin reduced coenzyme Q10 expression and mevalonate reversed the defect in INS-1 cells.”

now reads:

“Atorvastatin reduced COQB10 expression and mevalonate reversed the defect in INS-1 cells.”

In the Results, subsection ‘Atorvastatin reduced COQB10 expression and mevalonate reversed the defect in INS-1 cells’,

“In order to elucidate if increased oxidative stress and related disorders were due to a reduced expression of coenzyme Q10, we also measured the levels of this protein in INS-1 cells chronically (24 or 48 h) treated with atorvastatin (10 or 100 ng/mL). As shown in the Panels A and C of Fig. 9, our results showed a significant decrease in ubiquinone expression in atorvastatin-treated cells compared with control cells, as evaluated by Western Blot analysis.”

now reads:

“In order to elucidate if increased oxidative stress and related disorders were due to a reduced expression of coenzyme Q10, we also measured the levels of COQ10 protein, that functions in the correct activity of CoQ10 as electron carrier in the mitochondrial electron transport chain. In particular, we measured COQ10B protein expression in INS-1 cells chronically (24 or 48 h) treated with atorvastatin (10 or 100 ng/mL). As shown in the Panels A and C of Fig. 9, our results showed a significant decrease in COQ10B expression in atorvastatin-treated cells compared with control cells, as evaluated by Western Blot analysis.”

In the same subsection,

“On the basis of this evidence, and in order to determine whether this reduction primarily resulted from the inhibition of the mevalonate pathway, we studied the expression of coenzyme Q10 after the addition of increasing concentrations of mevalonate (50, 100, 500 or 1000 μM) to INS-1 cells contemporarily treated with 100 ng/mL atorvastatin for 48 h. As evidenced by Western Blot analysis, mevalonate increased the expression of ubiquinone with a statistically significant effect at the concentration of 500 μM (Fig. 9, Panel B and D).”

now reads:

“On the basis of this evidence, and in order to determine whether this reduction primarily resulted from the inhibition of the mevalonate pathway, we studied the expression of COQ10B protein after the addition of increasing concentrations of mevalonate (50, 100, 500 or 1000 μM) to INS-1 cells contemporarily treated with 100 ng/mL atorvastatin for 48 h. As evidenced by Western Blot analysis, mevalonate increased the expression of COQ10B with a statistically significant effect at the concentration of 500 μM (Fig. 9, Panel B and D).”

In Figure 9, all instances of “CoQ10” were changed to “COQ10B”. In the legend of Figure 9,

“Effect of exposure to atorvastatin and co-treatment with mevalonate on protein expression of coenzyme Q10 (CoQ10) in INS-1 cells. Panel A: representative Western blots for CoQ10 and Actin in control cells and in cells that had been in parallel exposed to atorvastatin 10 or 100 ng/mL of atorvastatin for 24 or 48 h. The cropped blots were run under the same experimental conditions. The full-length blots are presented in Supplementary Figure 1; Panel C: corresponding densitometric analysis; Panel B: representative Western blots for CoQ10 and Actin in control cells and in cells that had been in parallel exposed to atorvastatin 100 ng/mL and mevalonate at the reported concentrations for 48 h.”

now reads:

“Effect of exposure to atorvastatin and co-treatment with mevalonate on protein expression of COQ10B in INS-1 cells. Panel A: representative Western blots for COQ10B and Actin in control cells and in cells that had been in parallel exposed to atorvastatin 10 or 100 ng/mL of atorvastatin for 24 or 48 h. The cropped blots were run under the same experimental conditions. The full-length blots are presented in Supplementary Figure 1; Panel C: corresponding densitometric analysis; Panel B: representative Western blots for COQ10B and Actin in control cells and in cells that had been in parallel exposed to atorvastatin 100 ng/mL and mevalonate at the reported concentrations for 48 h.”

In the Discussion,

“The pivotal point of our results is, instead, the mitochondria; in pancreatic β-cells, with low antioxidant capacities, atorvastatin decreased CoQ10 levels and induced high oxidative stress, responsible for mitochondrial deterioration.”

now reads:

“The pivotal point of our results is, instead, the mitochondria; in pancreatic β-cells, with low antioxidant capacities, atorvastatin decreased COQ10B protein levels and induced high oxidative stress, responsible for mitochondrial deterioration.”

Further in the same section,

“Our data also evidenced that chronic exposure to atorvastatin reduced the expression of ubiquinone in pancreatic β-cell. Coenzyme Q10 is a product of the mevalonate pathway and in addition to constituting an important electron transporter of the mitochondrial respiratory chain, it is the only lipid-soluble antioxidant normally synthesized by the organism^46^. The role of CoQ10 protein depletion in our system is critical by virtue of the low anti-oxidative capacities of β-cells. Consequently, reduction of CoQ10 by atorvastatin, in a model where ROS-detoxifying constituents are already low, leads to increased generation of ROS which ultimately results in deterioration of the mitochondrial respiratory function^47^.”

now reads:

“Our data also evidenced that chronic exposure to atorvastatin reduced the expression of COQ10B protein in pancreatic β-cell. Coenzyme Q10 is a product of the mevalonate pathway and in addition to constituting an important electron transporter of the mitochondrial respiratory chain, it is the only lipid-soluble antioxidant normally synthesized by the organism^46^. The role of CoQ10B protein depletion in our system is critical by virtue of the low anti-oxidative capacities of β-cells. Consequently, the potential reduction of CoQ10 functional activity, in a model where ROS-detoxifying constituents are already low, leads to increased generation of ROS which ultimately results in deterioration of the mitochondrial respiratory function^47^.”

Finally, also in the Discussion,

“Together, these data demonstrated the existence of a link between statin-secondary mevalonate depletion, ROS accumulation and β-cell dysfunction, suggesting that the decrease in CoQ10 level and the consequent high oxidative stress, in a limited ROS-scavenging system, could be the triggering factor inducing mitochondrial dysfunction and down-regulation of insulin secretion.”

now reads:

“Together, these data demonstrated the existence of a link between statin-secondary mevalonate depletion, ROS accumulation and β-cell dysfunction, suggesting that the potentially decreased functional activity of CoQ10 and the consequent high oxidative stress, in a limited ROS-scavenging system, could be the triggering factor inducing mitochondrial dysfunction and down-regulation of insulin secretion.”

These changes do not affect the overall conclusions of the study. The errors have now been corrected in the PDF and HTML versions of the Article.

