# Supplementary material for: Atorvastatin but Not Pravastatin Impairs Mitochondrial Function in Human Pancreatic Islets and Rat β-Cells. Direct Effect of Oxidative Stress
Source: Sci Rep. 2017 Sep 19;7:11863. doi: 10.1038/s41598-017-11070-x (PMC5605712; doi:10.1038/s41598-017-11070-x)
Supplement: Supplementary file 1 — Supplementary Information [file 41598_2017_11070_MOESM1_ESM.pdf]

Supplementary Information for:

Atorvastatin but Not Pravastatin Impairs Mitochondrial Function in Human Pancreatic Islets and Rat β-Cells. Direct Effect Of Oxidative Stress

Francesca Urbano PhD[1], Marco Bugliani PhD[2], Agnese Filippello PhD[1], Alessandra Scamporrino PhD[1], Stefania Di Mauro PhD[1], Antonino Di Pino MD[1], Roberto Scicali MD[1], Davide Noto MD[3], Agata Maria Rabuazzo MD[1], Maurizio Averna MD[3], Piero Marchetti MD PhD[2], Francesco Purrello MD[1] and Salvatore Piro MD PhD[1].*

[1]*Department of Clinical and Experimental Medicine, Garibaldi Hospital, University of Catania, Catania, Italy*

[2]*Department of Clinical and Experimental Medicine, Islet Cell Laboratory, University of Pisa, Pisa, Italy*

[3]*Department of Biomedicine, Internal Medicine and Medical Specialties (DIBIMIS), University of Palermo, Palermo, Italy*

***Corresponding author**

Francesco Purrello, MD, Department of Clinical and Molecular Biomedicine, University of Catania, Internal Medicine, Garibaldi Hospital, Via Palermo, 636 - 95122 Catania, Italy,
Fax number: +39-0957598421, Phone number: +39-0957598401
E-mail: fpurrell@unict.it

This file includes:

- **Supplementary Table 1:** Clinical characteristics of human pancreatic islet donors;
- **Supplementary Figure 1:** Full blot images for the cropped western blot films showed in the Figures 4, 5, 7, 9 and 10.

Supplementary Table 1

	Age Years	Gender	BMI (Kg/m^2)	Cause of Death
**Donors**				
**Donor #1**	66	F	24.4	CVD
**Donor #2**	90	F	25.0	CVD
**Donor #3**	54	M	20.1	CVD
**Donor #4**	45	F	20.0	CVD
**Donor #5**	68	F	25.4	CVD
**Donor #6**	79	M	27.7	CVD
**Donor #7**	83	M	24.5	TRAUMA
**Donor #8**	75	M	22.9	CVD
**Donor #9**	59	M	25.9	CVD

(CVD: cardiovascular disease)

Supplementary Figure 1

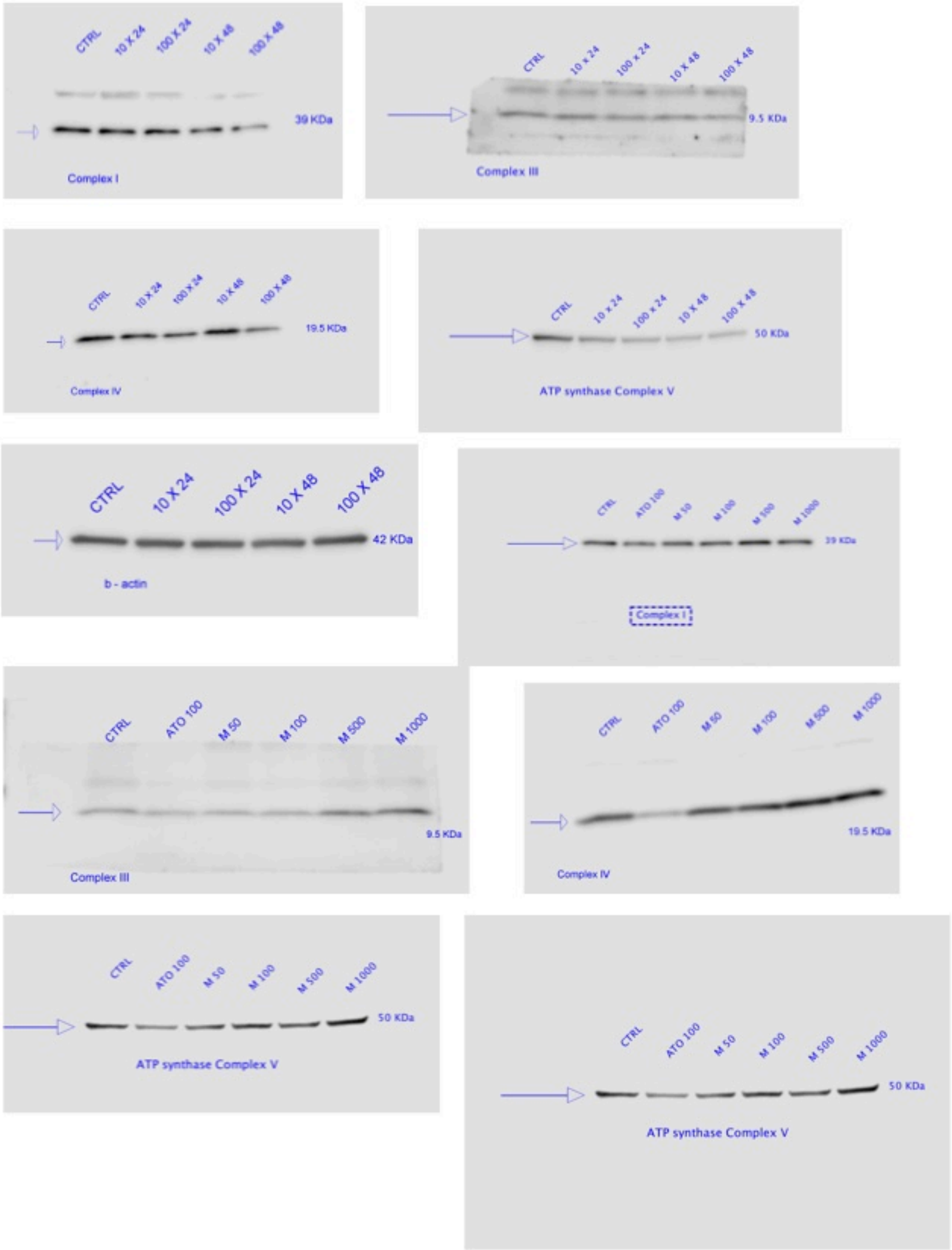

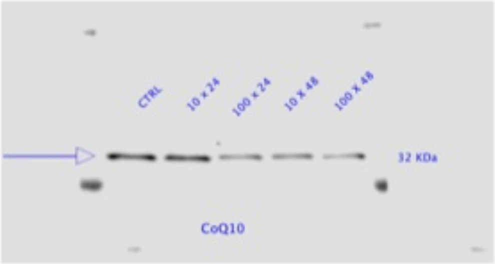
CTRL
10 x 24
100 x 24
10 x 48
100 x 48
32 KDa
CoQ10

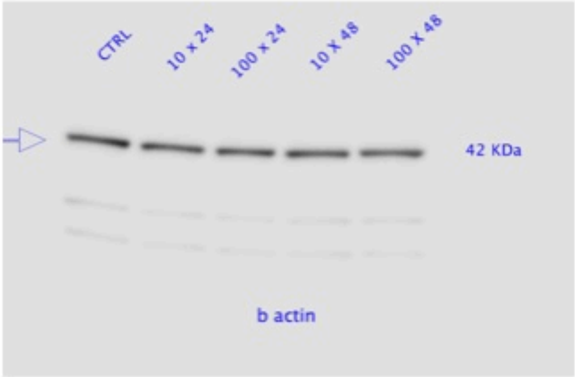
CTRL
10 x 24
100 x 24
10 X 48
100 X 48
42 KDa
b actin

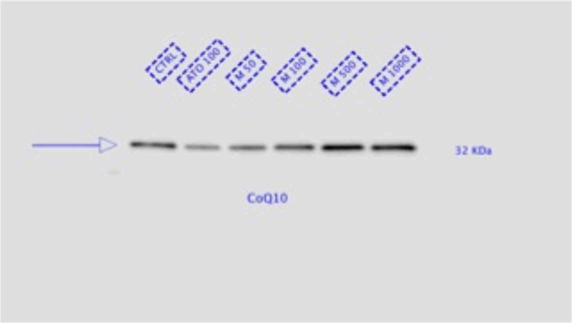
CTRL
ATO 100
M 50
M 100
M 500
M 1000
32 KDa
CoQ10

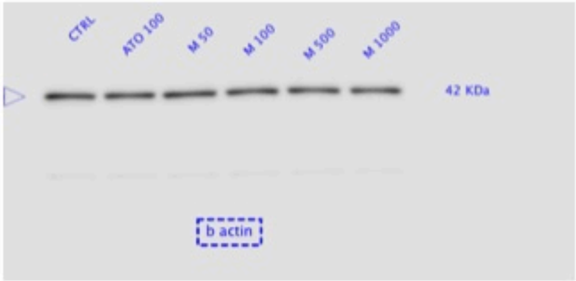
CTRL
ATO 100
M 50
M 100
M 500
M 1000
42 KDa
b actin

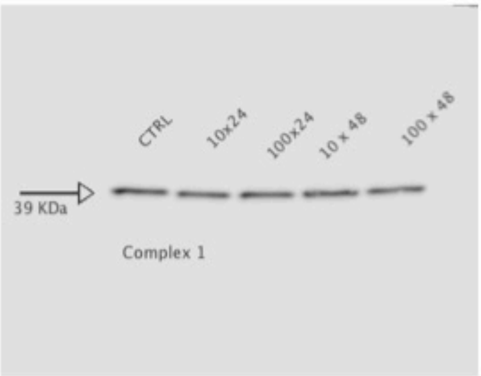
CTRL
10x24
100x24
10 x 48
100 x 48
39 KDa
Complex 1

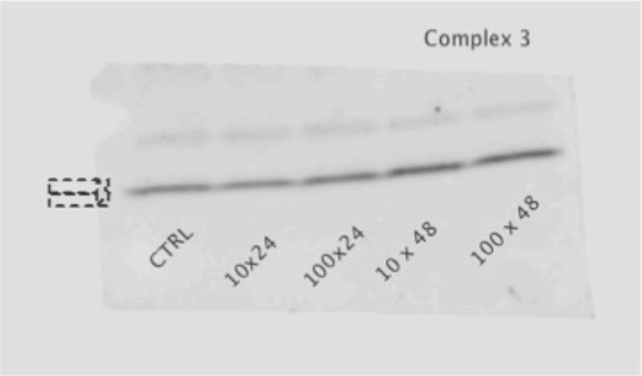
Complex 3
CTRL
10x24
100x24
10 x 48
100 x 48

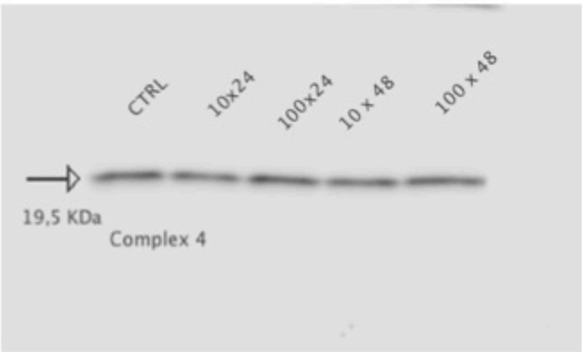
CTRL
10x24
100x24
10 x 48
100 x 48
19,5 KDa
Complex 4

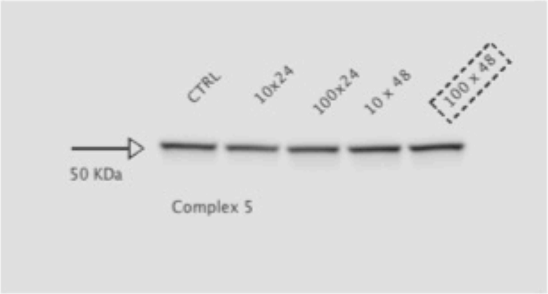
CTRL
10x24
100x24
10 x 48
100 x 48
50 KDa
Complex 5

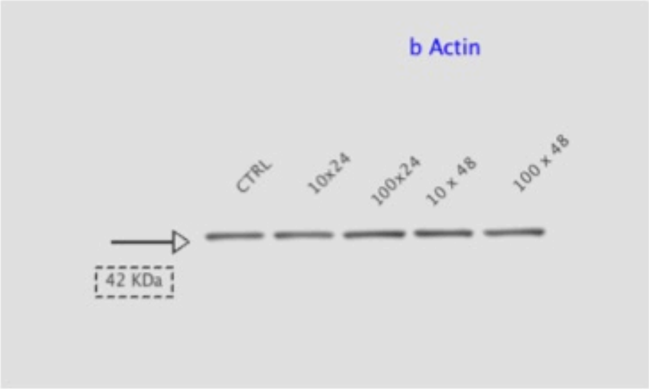
b Actin
CTRL
10x24
100x24
10 x 48
100 x 48
42 KDa

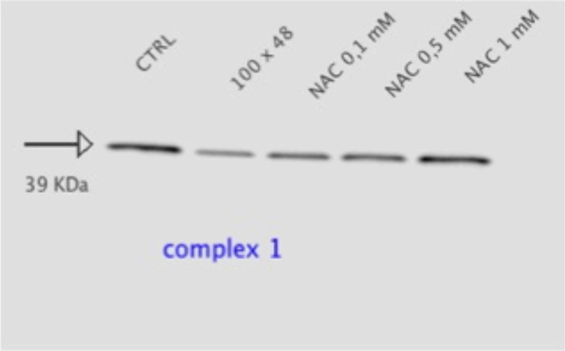
CTRL
100 x 48
NAC 0,1 mM
NAC 0,5 mM
NAC 1 mM
39 KDa
complex 1

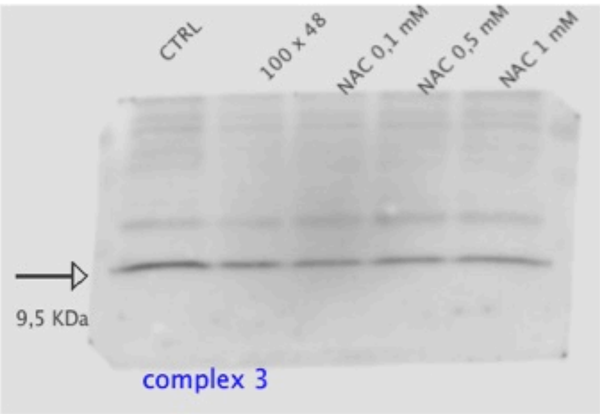
CTRL
100 x 48
NAC 0,1 mM
NAC 0,5 mM
NAC 1 mM
9,5 KDa
complex 3

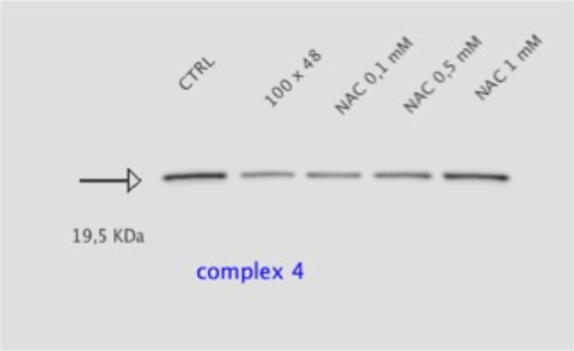
CTRL
100 x 48
NAC 0,1 mM
NAC 0,5 mM
NAC 1 mM
19,5 KDa
complex 4

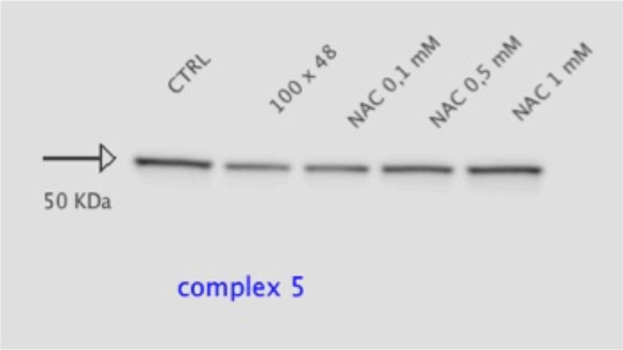
CTRL
100 x 48
NAC 0,1 mM
NAC 0,5 mM
NAC 1 mM
50 KDa
complex 5

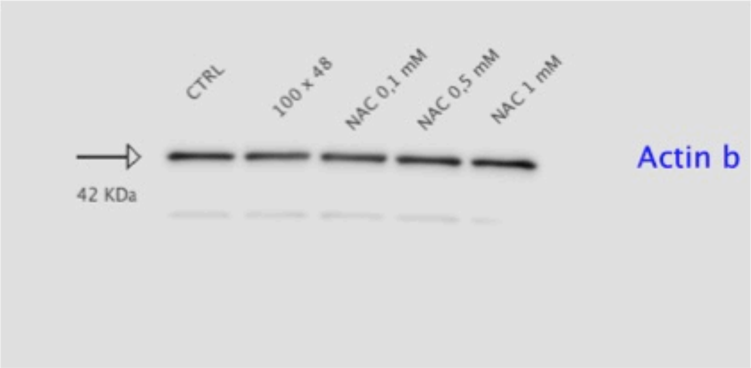
CTRL
100 x 48
NAC 0,1 mM
NAC 0,5 mM
NAC 1 mM
Actin b
42 KDa
